# Physical Forces May Cause the HoxD Gene Cluster Elongation

**DOI:** 10.3390/biology6030032

**Published:** 2017-06-23

**Authors:** Spyros Papageorgiou

**Affiliations:** Institute of Biosciences and Applications, National Center for Scientific Research “Demokritos”, 153 10 Athens, Greece; spapage@bio.demokritos.gr; Tel.: +30-210-895492

**Keywords:** Hox gene collinearity, vertebrates, HoxD cluster elongation, morphogen gradient

## Abstract

Hox gene collinearity was discovered be Edward B. Lewis in 1978. It consists of the Hox1, Hox2, Hox3 ordering of the Hox genes in the chromosome from the telomeric to the centromeric side of the chromosome. Surprisingly, the spatial activation of the Hox genes in the ontogenetic units of the embryo follows the same ordering along the anterior-posterior embryonic axis. The chromosome microscale differs from the embryo macroscale by 3 to 4 orders of magnitude. The traditional biomolecular mechanisms are not adequate to comprise phenomena at so divergent spatial domains. A Biophysical Model of physical forces was proposed which can bridge the intermediate space and explain the results of genetic engineering experiments. Recent progress in constructing instruments and achieving high resolution imaging (e.g., 3D DNA FISH, STORM etc.) enable the assessment of the geometric structure of the chromatin during the different phases of Hox gene activation. It is found that the mouse HoxD gene cluster is elongated up to 5–6 times during Hox gene transcription. These unexpected findings agree with the BM predictions. It is now possible to measure several physical quantities inside the nucleus during Hox gene activation. New experiments are proposed to test further this model.

## 1. Introduction

Edward B. Lewis discovered in 1978 that some genes (coined later Hox genes) located in sequence on the chromosome in the cell nucleus are expressed in the same order along the Anterior/Posterior (A/P) axis of the developing embryo [1]. This correlation is called spatial Hox gene collinearity and since then it is intensively studied. This collinearity is remarkable because it connects geometric entities differing by about four orders of magnitude: at the microscale the size of a Hox cluster is variable with minimum about 100 nm (See Section 4). At the macroscale the size of a multicellular early embryo is of the order 1 mm [2]. 

Lewis originally noticed the collinearity in the *Drosophila* embryo but it was later found that this property is observed in many other animal clades, e.g., vertebrates [3]. The evolution of Hox genes is investigated in depth and it is believed that an ancestral ur-Hox gene was in tandem duplicated and several Hox gene clusters were formed in different clades [4,5]. The structure of the different clusters varies from species to species and the vertebrates possess well organized and compacted clusters [3]. It is therefore the vertebrates, and in particular mice and chicks, that are extensively studied and several models are proposed to explain the accumulated experimental findings of Hox gene transcription. 

Besides spatial collinearity, another collinearity has been observed particularly in vertebrates: the first Hox gene (Hox1) in the sequence along the chromosome is expressed first followed later by the second Hox gene (Hox2) in the time sequence Hox1, Hox2, Hox3 etc. This important collinearity is coined temporal collinearity [3]. Temporal collinearity is crucial in evolution and it is considered that it is a major factor in keeping Hox genes together as observed in the vertebrates [3].

**Figure 1:**
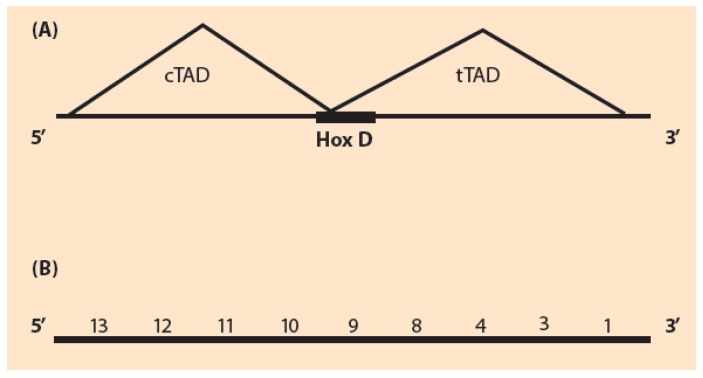
HoxD cluster topology. (**A**) Schematic representation of the HoxD gene cluster D flanked by the telomeric (tTAD) and the centromeric (cTAD); (**B**) The gene order of the complete HoxD cluster: (Hoxd1, Hoxd3, Hoxd4, Hoxd8, Hoxd9, Hoxd10, Hoxd11, Hoxd12, Hoxd13).

In order to explain Hox gene collinearity, a model was put forward by Duboule and collaborators [6,7]. This established model, called “two-phases model” (T-PM), is relying on standard bio-molecular mechanisms. T-PM consists of an early and a late phase [7]. In the early phase a positive influence from the telomeric side is balanced by a centromeric repression. A late phase is determined by local regulatory elements. The T-PM correctly describes the results of many genetic engineering manipulations consisting of a deletion (or duplication) of Hoxd genes in the HoxD cluster [7]. In a deletion, the expressions of the remaining genes anterior or posterior to the deleted genes are usually strongly affected. The study of the mutant embryo Hox gene expressions provides valuable information. The comparison to the wild type expressions can elucidate many sides of the mechanism of Hox gene activation. In another experimental set up instead of deletions, Hoxd duplications were performed [7]. The T-PM relies on the long-range regulatory elements enhancing expression of Hox genes within the distinct topologically associating domains (TAD) located at the telomeric and the centromeric sides of the Hox cluster [8,9]. With the new genomic technologies it is possible to study the three-dimensional chromatin organization of the TADs [10]. Their evolutionary stability across different vertebrates and invertebrates is indicative of their crucial interactions with the neighboring genome. The size of the TADs is large of the megabaze-order [10]. The HoxD cluster is located at the interface of the two TADs (Figure 1A). 

## 2. The Biophysical Model

**Figure 2:**
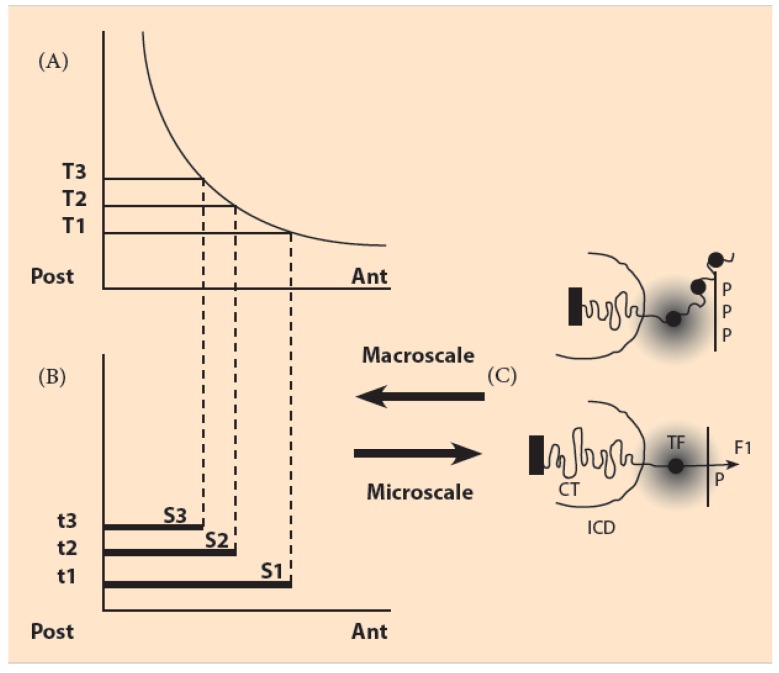
The macroscale morphogen gradient and the microscale Hox gene clustering in space and time (adapted from Almirantis Y., Provata A. and Papageorgiou S. [11]. (**A**) Concentration thresholds (T1, T2, T3) divide the A/P axis in partially overlapping expression domains; (**B**) The time sequence (t1, t2, t3) combined with the threshold sequence (T1, T2, T3) determine the Hox1, Hox2, Hox3 activation in space and time. S1, S2, S3 are the partially overlapping and nested expression domains of Hox1, Hox2, Hox3 (**C**) (bottom) In an anterior cell of S1, a small force F1 pulls Hox1 (black spot) out of the CT toward the ICD in the regime of the TF (grey domain). Apposition of molecule P opposite the telomeric end of HoxD cluster. (top) At a later stage in a more posterior location of S3, a stronger force pulls Hox1, Hox2, Hox3 out of the CT. Apposition of PPP molecules.

A quite different approach is followed in another explanatory model, the “biophysical model” (BM). This model is based on the hypothesis of physical forces generated and acting on the telomeric side of the Hox cluster. The forces are pulling sequentially the Hox genes to a domain where transcription is possible. Motivation for this approach is the multiscale nature of Hox collinearity (Figure 2). Basic laws of Physics rather than biomolecular mechanisms are more adequate to bridge phenomena (or material bodies) in different scales. For example in the simple hydrogen atom the long-range Coulomb force holds the electron cloud around the nucleus (proton). Fortuitously, the size of the electron orbitals are bigger than the atomic nucleus by about 4 orders of magnitude.

The BM was initially proposed in 2001 but its adoption was limited because the experimental evidence was not sufficient to confirm such forces [12]. According to the BM when the HoxD cluster is inactive, the Hox genes are compacted inside the chromatin territory (CT). When the pulling forces are generated the genes are relocated sequentially, starting with Hoxd1, to a region inside the interchromosome domain (ICD) in the area of the transcription factory (TF) where transcription is possible (Figure 2C). TFs are intensively studied since they are the important nuclear sites towards which DNA moves for transcription [13]. 

A heuristic simple force F (whose measure is F) was introduced which depends on two factors “P” and “N”.

F = P × N(1)

In the microscale, N represents the total “negative charge” uniformly distributed in the Hox cluster. Τhe “positive charge” P is located in the CT opposite to N and reflects the position of the particular cell along the macroscopic anterior/posterior (A/P) embryonic axis (Figure 2) [14,15] (see next Section). The cell position is determined by the morphogenetic gradient values: anterior position: P small-posterior position: P big. The pulling force F increases causing the sequential extrusion of the Hoxd genes in the order Hoxd1, Hoxd2, Hoxd3 etc. The simple Equation (1) was applied to simulate the genetic engineering experiments of deletions (or duplications) for which the factor N varies. Surprisingly, the experimental results were correctly reproduced by Equation (1) [14,15,16,17].

It is instructive to consider an example of interdependence of the factors P and N in Equation (1). In an experiment of “posterior deletion” the deleted Hoxd genes are posterior to the probe gene. In such an experiment on the mouse limb, the wild type expression of the probe gene is modified [6]: firstly it appears later than normally and secondly the spatial extent of the expression is limited to the posterior region. This is surprising according to the T-PM but it is explained following the BM and Equation (1): the posterior Hoxd gene deletions decrease the value of the N-factor in Equation (1). In order to recover the value of the pulling force F, so that the probe gene is properly extruded, P must increase. According to the BM a rule is formulated for the deletions: posterior deletions cause a retarded posteriorization of the probe gene while anterior deletions cause premature anteriorization of the probe gene [15]. An analogue rule holds for gene duplications. These properties reflect a natural interlocking of space and time as a result of the BM (see Figure 2). This entanglement is in agreement with the observed spatial and temporal collinearity of the Hoxb expressions in the early chick embryo [18]. In a recent experiment in the early *Xenopus* embryo it was found that Hox temporal collinearity is “indispensable” in generating spatial collinearity [19]. Furthermore, it was noticed that the above entanglement was dismantled at later stages of the chick embryo development [18].

**Figure 3:**
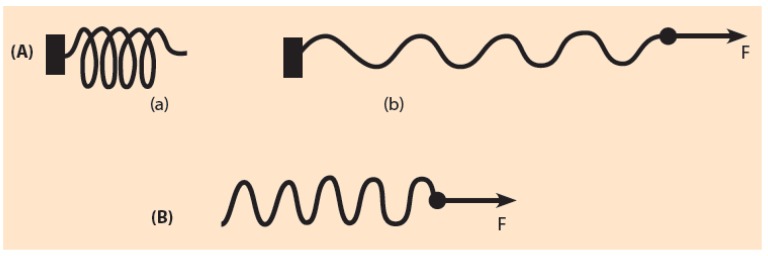
Elastic spring expansion. (**A**) (a) An uncharged elastic spring with fixed left end and free right end; (b) The pulling force F elongates the spring in the direction of the force; (**B**) The fixed end of the spring is cut-off. The same force F causes: (1) a slide to the right of the loose spring; (2) an elongation of the loose spring. This elongation is smaller than the elongation of Figure 3A(b).

In the BM, the HoxD cluster is schematically represented by an elastic spring with its free end located at the telomeric end of the Hox cluster [15,16]. The other end of the spring is firmly fixed and attached to the neighboring chromatin domain centromeric to the Hox cluster (Figure 3Aa). This centromeric domain beyond Hoxd13 incorporates probably the Evx2 gene. The pulling force is ever increasing in the telomeric-centromeric direction causing a spring elongation which obeys Hooke’s law of elastic expansion (Figure 3A(b)). The spring representation is a mechanistic analogue resembling the HoxD cluster response to genetic engineering manipulations. In a Hoxd deletion the chromosome fiber continuity is restored: the telomeric and centromeric ends of the remaining Hox cluster are connected to the two ends of the flanking DNA fiber. The above spring analogue of the HoxD cluster is an oversimplification since the 3D organization of the Hox genes and their interactions with the regulatory elements located in the flanking TADs are quite complex. However, the BM with the oversimplification of the elastic spring can surprisingly explain the above experiments [16].

## 3. Morphogen Gradient Experiments

In addition to the above experimental sets at the microscale genetic level, a different class of experiments have been performed involving the macroscale component of the BM: at the macroscopic level, morphogenetic gradients are established along the 3 axes of the embryo (Figure 2A) [20,21]. Examples of morphogenetic signals are the Sonic hedgehog (Shh) and the Fiber Growth Factors (FGF) cooperating in the limb development [22]. The signals from these gradients are transduced to the microscale level inside the cell nucleus [23]. The apposition of such transduced molecules at specific locations inside the nucleus has been studied as in the case of SMAD2 [24] or the DSH protein accumulation in the xenopus cell nucleus [25]. The apposition and concentration of these molecules, reflecting the cellular position on the morphogen gradient, may determine the P-factor in Equation (1). It turns out that passive diffusion, combined with first order chemical kinetics, is the principal mechanism in the creation of the monotonic morphogen gradients in the A-P axis [26,27]. In an alternative hypothesis, a time space translation mechanism could initiate this axis formation. This proposed mechanism can account for temporal collinearity [28]. 

Numerous experiments have been performed where the morphogen source is modified and accordingly the gradient is changed. For instance, when the morphogen source is removed at the apical ridge of the limb bud the gradient fades out and the expression of the Hoxa13 switches off while the Hoxa13 expression is rescued if an FGF soaked bead is implanted in place of the ridge [26]. Further concentration increase of the implanted FGF bead causes an increase of the morphogen gradient. This increase is necessary for the change of the spatial limits of expression of Hoxa13. It is remarkable that, when an FGF4 bead is implanted at the tip of an intact bud, the Hoxa13 expression is unexpectedly inhibited around the tip [26]. This can be explained by the gradient model if it assumed that the Hoxa13 expression is allowed within a precise FGF concentration range between a lower and an upper threshold [26]. In the wild type bud, the FGF4 concentration at the tip is below the upper threshold. In contrast, when the FGF4 bead is implanted at the tip of the bud the morphogen concentration exceeds the upper threshold. Recent studies have shown that, for signaling along the proximo-distal axis, a timing mechanism dependent on histone acetylation status is also necessary [20,27].

The case of cooperating morphogens Shh and FGF has been worked out both analytically and numerically in the limb bud. The bud was approximated by a three-dimensional orthogonal parallelepiped and the estimated Hoxd expressions were in agreement with the expressions observed up to that time [20]. Recently, new experiments were performed where cultured limb cells responded to the cooperating Shh and FGF signals and the Hoxd11, Hoxd12 and Hoxd13 expressions were analyzed [22]. It was concluded that ZPA and AER signaling, although necessary, are not sufficient for the complete Hoxd gene activation.

The methods and techniques involved in all these experiments are essentially biomolecular and biochemical. The T-PM is suitable for a biomolecular interpretation. In contrast, the BM can only indirectly handle the above findings. The reason is that the BM relates mainly physical-geometric quantities of the genome and these quantities are not measured in the above experimental set-ups. For a direct implementation of the BM, new experimental methods are needed enabling the exact determination of the physical-geometric entities and their variations inside the cell nucleus. Such entities are chromatin fiber elongations, chromatin bending, twisting etc.

## 4. New Methods and the Biophysical Model

During the last decade or so, new techniques were developed making possible the 3D DNA FISH analysis of the chromatin organization in vivo. The techniques include superresolution imaging-STORM (stochastic optical reconstruction microscopy). These novel methods were applied to experiments on the mouse HoxD cluster during its transition from the inactive state to gradual gene transcription [8,9]. These experiments revealed some unexpected features of the physical-geometric structure of the HoxD cluster. For instance, it was observed that the HoxD cluster is variably elongated, depending on the stage of transcriptional activity. The cluster elongation was measured and it was found that the length of an elongated cluster can exceed 500 nm [9]. For example the size of the “inactive” HoxD cluster in the forebrain is about 100 nm while in the distal forelimb it exceeds 500 nm [9,29]. Furthermore, it was unexpectedly found that all domains of the HoxD cluster, activated and non-activated, are elongated [9,30]. Note that these new data are suitable to be compared to the BM expected results. It is therefore important to set-up experiments involving the measurement of other suitable physical-geometric quantities.

In the above spirit, a first case of directly testing the biophysical model is to anticipate the results from the elastic spring picture: before activation the HoxD cluster is compacted and represented by an uncharged spring (Figure 3A(a)). When a pulling force F is applied at the free end of the spring, the spring is elongated. In a simple mechanical spring, it is observed that the local elongations are the same in all domains of the spring. In this simple approximation, an increase of the applied force F causes a proportional spring elongation. As mentioned above, this mechanical picture is compatible with the observed elongation of the HoxD cluster. Local chromatin interactions, responsible for gene transcription, do not affect the cluster elongation. Therefore, the transcribing domains of the cluster are elongated as are the non-transcribing domains [8,9]. It is remarkable that the complicated intergene interactions have an overall result summarized in a simple mechanical analogue responding to physical forces. This response is independent of the transcriptional state of the Hoxd genes.

## 5. Some Predictions of the Biophysical Model

The above experiments are approximately interpreted by the BM. This is encouraging to apply the new methods to further test this model. To this end, some experiments are proposed below.

Deletions involving the centromeric domain flanking the HoxD cluster:

Consider first the free movement of a rigid body in the absence of friction. The application of a pulling force on this body will cause its slide (shift) along the direction of the force without causing a deformation of this body. Consider now a non-rigid body moving in the presence of friction. Consider further that this body is an elastic spring fixed at its left end as depicted in Figure 3A(a). The spring remains uncharged if no force acts on it. A force F applied on the loose end will cause a normal spring elongation En (Figure 3A(b)). What will happen if the fastened left end of the spring is cut off and the same force F is applied to the loose right end of the spring? According to simple Mechanics the following results are expected:

Prediction 1: the entire elastic spring will shift to the right. 

Prediction 2: the spring will be partly elongated (Figure 3B). This elongation Ed will be smaller than the elongation of Figure 3A(b) because a fraction of the pulling energy is dissipated in the spring slide:

Ed < En.

**Figure 4:**
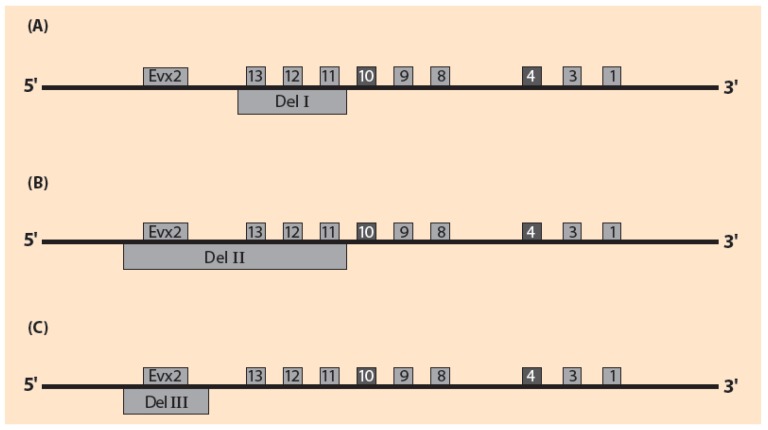
Schematic representation of the Kondo-Duboule deletion experiments on HoxD cluster. Hoxd10 and Hoxd4 are probe genes; (**A**) In Del I the posterior Hoxd11, Hoxd12, Hoxd13 are deleted; (**B**) In Del II, besides Hoxd11, Hoxd12, Hoxd13, the centromeric flanking domain (including Evx2) is also deleted; (**C**) In Del III the centromeric flanking domain (including Evx2) is deleted.

It is challenging to explore if the above mechanical spring picture can be extended to other genetic engineering experiments on the HoxD cluster. Motivation for this proposition is the result of two experiments (I,II) performed by Kondo and Duboule on the mouse embryo several years ago [31]. In experiment I the posterior Hoxd genes (Hoxd11, Hoxd12, Hoxd13) were deleted (Figure 4A). In experiment II, besides the above deletions, the flanking centromeric domain containing Evx2 was also deleted: (Evx2, Hoxd13, Hoxd12, Hoxd11) (Figure 4B). At stage E8 of the wild type embryo, the probe genes Hoxd10 and Hoxd4 expressions start appearing. In experiment I, these expressions, unexpectedly, do not appear [31]. In experiment II, the expressions of Hoxd10 and Hoxd4 appear prematurely. These results were considered surprising [31]. Furthermore, they are indicating that the deletion of the centromeric region (Evx2 included) has a drastic effect on the HoxD cluster activation.

According to the BM the above results can be explained as follows: 

In experiment I, according to the rule formulated in Section 2, a posterior deletion causes a retarded posteriorization of a probe gene (Figure 4A). Therefore, the expressions of Hoxd10 and Hoxd4 have not been observed because these expressions have not yet appeared at stage E8.

In experiment II the premature appearance of the Hoxd10 and Hoxd4 expressions, indicates that the deletion of the flanking centromeric region (Evx2 included) strongly overrules the retardation of experiment I (Figure 4B).

If the spring analogy could be extended to these manipulations, the deletion of the centromeric region only would correspond to the cut off of the fastened end of the spring (Figure 4C). Therefore, a force F, as in Figure 3B, would slide the HoxD cluster anteriorily. The deletion of this centromeric region (including Evx2) as in Figure 4C would lead to the following predictions: (a) Shift of the HoxD cluster anteriorily, leading to a premature expression of the probe genes. (b) The elongation of the mutant HoxD cluster at any stage is smaller than the wild type elongation at the same stage. 

As stressed before, the above deletions are complex processes involving the removal of several regulatory elements whose effect on the Hoxd expressions is unknown. It is therefore a daring hypothesis to expect a final simple outcome like Predictions (a) and (b) for the HoxD cluster although the detailed deletion effects are complex and cannot be anticipated. Therefore, it is worth performing the deletion experiment of Figure 4C because the confirmation of the above Predictions would broaden the validity range of the BM and its spring analogy.

## 6. Conclusions

The simple Equation (1) incorporates in a compact form both the macroscale component (P) and the microscale component (N) of Hox gene collinearity. Furthermore this equation can explain the separate experimental set-ups at the different scales: the morphogen gradient variations on one hand and the genetic engineering in Hox clusters on the other. As stressed in Section 2 and Section 3, at the early embryonic stages Equation (1) entails the observed time-space entanglement [19,28].

An important question was raised: does the separation of active from non-active Hox genes “underlie collinear activation or is a consequence of it” [32]. According to the BM the physical separation of Hox genes and their collinear activation are indispensable and non-separable elements of a single mechanism [33,34]. This mechanism incorporates physical forces acting on the DNA fiber and translocating the Hox genes. The gene translocation is followed by gene transcription [33,34]. A natural consequence is to propose a synthesis of the models BM and T-PM into a single integrated mechanism in two steps for the vertebrate Hox gene activation. In the first step the BM translocates the Hox genes in the right position in the ICD. In the second step the T-PM organizes the transcription of the translocated genes. It is remarkable that, following a quite different approach, a similar proposition is put forward by Fabre et al.: “the structural organization of the HoxD gene cluster predates transcription” [9,30]. The convergence of the two conclusions is very satisfactory but supplementary experiments are needed to consolidate the mechanism in two steps for the activation of Hox genes.

In the last decade, diversifying aspects of Hox gene activity have been intensively studied. For example, significant progress was reported on the Hox gene regulatory network of the hindbrain segmentation in vertebrates [35]. However, in this field there are still many open questions to be answered. For instance how the lamprey at the origin of the vertebrate phylogenetic tree possesses nested Hox gene expressions which may be coupled to hindbrain segmentation [36]. If the Biophysical Model comes into play, it may help in this fascinating quest. 

## Abbreviations

The following abbreviations are used in this manuscript:

A/P	Anterior-posterior
BM	Biophysical model
CT	Chromatin territory
FGF	Fiber growth factor
ICD	Interchromosome domain
Shh	Sonic hedgehog
TAD	Topological associating domain
TF	Transcription factory
T-PM	Two-phases model
